# Greater distance from the glenosphere center to the acromion reduces risk of acromial impingement in semi-inlay reverse shoulder arthroplasty

**DOI:** 10.1016/j.jseint.2024.05.007

**Published:** 2024-05-28

**Authors:** Itaru Kawashima, Norimasa Takahashi, Keisuke Matsuki, Hisato Watanabe, Ryo Haraguchi, Hayato Ryoki, Kenji Kitamura, Thomas W. Wright, Scott A. Banks

**Affiliations:** a Department of Orthopaedic Surgery and Sports Medicine, University of Florida, Gainesville, FL, USA; b Department of Orthopaedic Surgery, Nagoya University Graduate School of Medicine, Nagoya, Aichi, Japan; c Sports Medicine and Joint Center, Funabashi Orthopaedic Hospital Funabashi, Chiba, Japan; d Department of Mechanical & Aerospace Engineering, University of Florida, Gainesville, FL, USA

**Keywords:** Reverse total shoulder arthroplasty, Subacromial impingement, Acromial impingement, Acromial fracture, DA, DGT, Shoulder arthroplasty

## Abstract

**Background:**

Recently, the issue of subacromial notching, caused by acromial impingement has been reported. The purpose of this study was to assess the impact of differences in the distance between the glenosphere center and the greater tuberosity (DGT) and the distance between the glenosphere center and the acromion (DA) on the closest distance between the greater tuberosity and the acromion during active abduction in shoulders with reverse total shoulder arthroplasty (RSA).

**Methods:**

Eleven shoulders with semiinlay RSA were analyzed. Subjects underwent fluoroscopy during active scapular plane abduction. Computed tomography of their shoulders was performed to create three-dimensional (3D) implant models at a mean of 16 months after surgery. Using model-image registration techniques, poses of 3D implant models were iteratively adjusted to match their silhouettes with the silhouettes in the fluoroscopic images (shape matching), and 3D kinematics of implants were computed. The closest distance between the acromion and greater tuberosity was computed at maximum abduction. DA and DGT were measured from 3D surface models. Shoulders were divided into two groups based on DA and DGT measurements and their closest distance data were compared between the groups.

**Results:**

There were 7 shoulders with DA ≥ DGT, and 4 shoulders with DA < DGT. Shoulders with DA ≥ DGT showed a significantly wider distance between the greater tuberosity and acromion at maximum abduction compared to those with DA < DGT (5.9 ± 2.4 mm vs. 0.6 ± 0.7 mm, respectively, *P* = .0021). There were no significant differences in maximum glenohumeral abduction angle and humeral abduction angle between the two groups. Although DA was significantly greater in shoulders with DA ≥ DGT than in those with DA < DGT (43.7 ± 4.4 mm vs. 35.1 ± 6.7 mm, respectively, *P* = .0275), there was no significant difference in DGT between the two groups.

**Conclusion:**

When DGT is less than DA in shoulders with RSA, the closest distance between the greater tuberosity and the acromion at maximum abduction is significantly wider compared to cases where DGT is greater than DA by 3D measurement. Therefore, acromial impingement is less likely to occur in shoulders with RSA when DA is greater than DGT. To avoid acromial impingement, it might be important to make DA greater than DGT.

Although reverse total shoulder arthroplasty (RSA) has a relatively short history, it has shown good results and the number of surgeries has tripled from 2007 to 2012.^6^ However, the procedure is not without complications. In medialized RSA, scapular notching, which is an erosive lesion on the inferior scapular neck due to impingement of the humeral implant, is often observed, and it can have an impact on clinical outcomes.^8^ Lateralized RSA works favorably by reducing the incidence of scapular notching and improving external rotation through the lateralization of the humerus.^18^ Yet, Jeong et al^3^ have reported that the lateralization of both glenoid and humerus side was correlated with subacromial notching, caused by acromial impingement, occurring in 12.8% of RSA cases and negatively affecting postoperative clinical outcomes. Therefore, surgeons may need to consider the appropriate amount of lateralization in RSA to minimize scapular neck notching and to improve external rotation while avoiding subacromial notching. However, the safe and optimal degree of lateralization in this context remains unclear, and there are no specific parameters or indicators available to avoid subacromial impingement or notching.

The previous study has reported predicting bony impingement using a preoperative planning model based on isolated glenohumeral motion.^20^ However, these findings are limited as they stem from a virtual-model-based study, and may not correlate with actual multifactorial clinical motion. Therefore, in vivo motion analysis appears to be necessary. There are various techniques for in vivo motion analysis. The three-dimensional (3D) - two-dimensional (2D) model-image registration method, which analyzes motions by superimposing 3D models of bones and implants on fluoroscopic images, enables highly accurate analysis.^8^ Kinematic analysis of RSA using a 3D-2D model-image registration method has been previously performed to quantify the risk of scapular neck notching.^15^ However, this same type of analysis has not been performed to assess risk factors for acromial impingement in shoulders with RSA.

Previous studies have reported an association between the distance from the humeral center of rotation to the greater tuberosity, and the distance from the humeral center of rotation to the acromion, with subacromial impingement.^10^ However, it remains unclear whether this phenomenon is applicable to shoulders with RSA, where the center of rotation becomes the glenosphere center. Our mechanistic model posits that the humerus, including the greater tuberosity, makes a circular movement around the glenosphere center. If the distance between the glenosphere center and the acromion (DA) is greater than the distance between the glenosphere center and the greater tuberosity (DGT), we expect the greater tuberosity will not impinge on the acromion.

Thus, the purpose of this in vivo kinematic study was to evaluate the DGT and the DA in shoulders with RSA, and to assess the impact of differences in DGT and DA on the closest distance between the greater tuberosity and the acromion during active abduction in shoulders with RSA. We hypothesized that when DGT is less than DA, the distance between the greater tuberosity and the acromion is wider compared to cases where DGT is greater than DA.

## Materials and methods

### Patient selection

This study was approved by the Institutional Review Board and Ethics Committee of our institution. Patients were included who underwent surgery using an uncemented semiinlay RSA (Medacta Shoulder System; Medacta International, Castel San Pietro, Switzerland) by an experienced shoulder surgeon at a single institution. A minimum of one year follow-up in the same institution was obtained between November 2022 and October 2023. Exclusion criteria included patients who did not consent, patients with complications and pseudoparalytic shoulders for they were thought to be less prone to acromial impingement.^21^

Patient records, including surgical reports were reviewed for preoperative demographic information (diagnosis, sex, age at surgery, body weight, height, body mass index), affected side, active range of motion, and American Shoulder and Elbow Surgeons (ASES) Standardized Shoulder Assessment Form score evaluated at one year after surgery.^19^

### Surgical intervention

All surgeries were performed by an experienced shoulder surgeon (N.T.) under general anesthesia with an interscalene block in the beach-chair position. The deltopectoral approach was utilized in all cases. After the subscapularis tendon was peeled off from the lesser tuberosity, the anterior capsule and subscapularis tendon were adequately separated, and the subscapularis tendon was mobilized to facilitate later repair. Subsequently, the capsule was resected from the humeral neck to expose the glenohumeral joint. Osteotomy of the humeral head was performed using a patient-specific guide. The uncemented semiinlay type RSA implant system (Medacta Shoulder System145°; Medacta International, Castel San Pietro, Switzerland) was used for surgery, employing a patient-specific implant guide and preoperative planning system. The implant system was categorized as a lateralized RSA, possessing an approximately intermediate level of lateralization according to the previous study.^22^ The humeral prosthesis was typically press-fitted to the same size as the preoperative plan. The glenoid baseplate was positioned with the target superior inclination ranging from −10 to 0°, using a patient-specific implant guide to prevent the glenosphere from facing upwards. The subscapularis tendon was repaired with a minimum of three tendon-to-bone stitches using high-strength sutures. Throughout the procedure, efforts were made to preserve the residual rotator cuff, except for the supraspinatus.

### Image acquisition

Fluoroscopic images of scapular plane abduction were recorded at a mean of 16 months (range, 12-27) after surgery (FLEXAVISION F4; Shimadzu, Kyoto, Japan). Images were acquired at 7.5 frames/s with 414 × 424-mm field of view and 1296 × 1328 image matrix. The patient stood with their torso at approximately 30° to the plane of the image intensifier, so that the scapula body was perpendicular to the x-ray beam.^11^ Scapular plane active abduction without any assistance was performed from arm at side to maximum abduction in approximately 5 seconds. During the activity, the elbow was fully extended, and the palm was directed forward (thumbs-up position). The body of the patient was not constrained to allow natural motion of the arm. The images taken at the maximum active abduction of the humerus were employed for the study.

The patients also underwent computed tomography scans of the shoulder (Alexion, Toshiba, Tochigi, Japan). The imaging parameters were as follows: slice pitch, 0.3 mm; image matrix 512 × 512; pixel size, 0.468 × 0.468. Iterative reconstruction techniques were used to minimize metal artifact.

### Three-dimensional models

3D surface models of scapular implants, encompassing the glenosphere, baseplate, and screws, were generated from computed tomography images using segmentation software (ITK snap; Penn Image Computing and Science Laboratory, Philadelphia, PA, USA).^23^ These models were created both with and without bones, including the distal clavicle and acromion. Similarly, humeral implants, both with and without the proximal humerus, were also generated using the same methodology. The modeling accuracy has been confirmed by deviation analysis with the root mean square error of 0.4 mm when comparing computed tomography derived models to the corresponding computer-aided design models.^17^ Anatomic coordinate systems were embedded using commercial software (Geomagic Wrap; 3D Systems, Rock Hill, SC, USA) according to previous reports.^14^^,^^17^ Computer-aided design models of polyethylene inserts were obtained from the manufacturer (Medacta International, Castel San Pietro, Switzerland). In brief, the origin of the humeral implant model was set at the center of the curvature of the polyethylene insert, and the origin of the scapular implant model was set at the center of the glenosphere. The X-axes of both models were set in the mediolateral direction, the Y-axes in the superoinferior direction, and the Z-axes in the anteroposterior direction.

### Model-image registration and data processing

**Figure 1:**
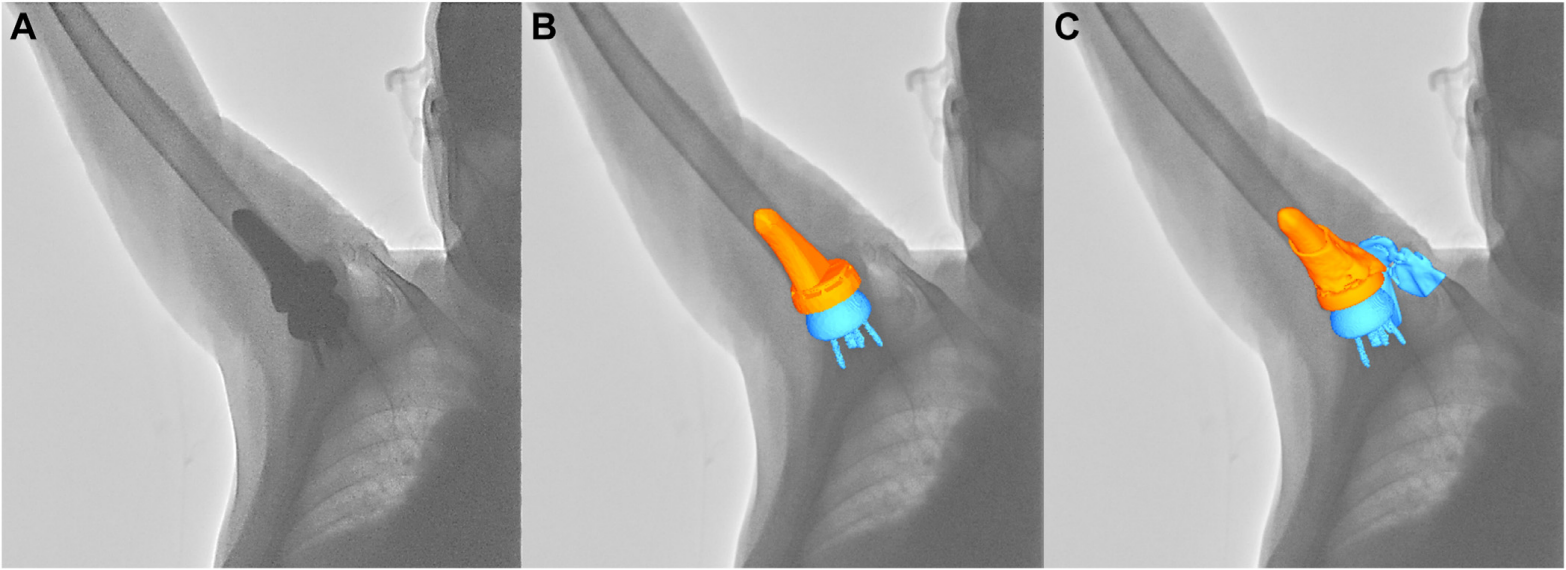
The three-dimensional to two-dimensional model-image registration for reverse shoulder arthroplasty. (**A**) A fluoroscopic image during scapular plane abduction. (**B**) Three-dimensional implant models were matched with the silhouette of the implants on the fluoroscopic image. (**C**) After matching three-dimensional implant with bone models.

The 3D position and orientation of models were determined using model-image registration techniques using an open source software program (JointTrack; University of Florida, Gainesville, FL, USA).^1^^,^^13^ The models were projected onto the fluoroscopic image, and their three-dimensional poses were iteratively adjusted to match their silhouettes with the silhouettes in the fluoroscopic image (Fig. 1). The accuracy of this matching method was 0.5mm for in-plane translation and 0.8° for in-plane rotation in a previous study.^16^

The implant poses relative to the x-ray coordinate system and the poses of a humeral implant relative to a glenosphere were determined using Cardan angles (z-x-y order).^11^ Abduction of humeral implant was defined as rotation about the humeral Z-axis, and internal/external rotation was defined as rotation about the humeral Y-axis. The poses of a glenosphere were defined as follows: forward/backward rotation about X-axis; internal/external rotation about Y-axis; upward/downward rotation about Z-axis. Glenohumeral abduction was defined as humeral kinematics relative to the glenosphere.

### Distance evaluation

**Figure 2:**
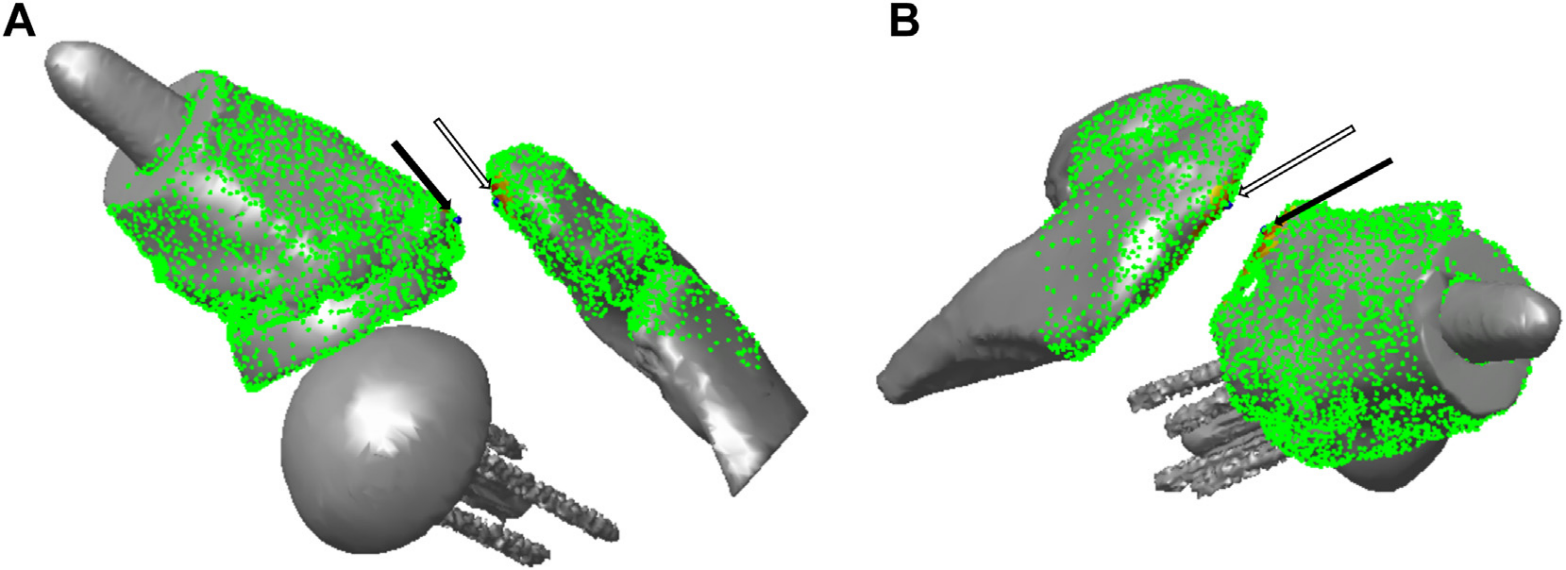
Calculating the closest distance between acromion (*white arrow*) and greater tuberosity (*black arrow*) based on model point clouds (MATLAB; Mathworks, Natick, MA, USA).

The closest distance between the acromion and proximal humerus was computed using the combined glenosphere, acromion and distal clavicle model and the humeral implant and proximal humeral model. The distance was computed at maximum abduction by finding the minimum distance from the scapular point cloud to the humeral point cloud (MATLAB; Mathworks, Natick, MA, USA) (Fig. 2).

### DA and DGT evaluation

**Figure 3:**
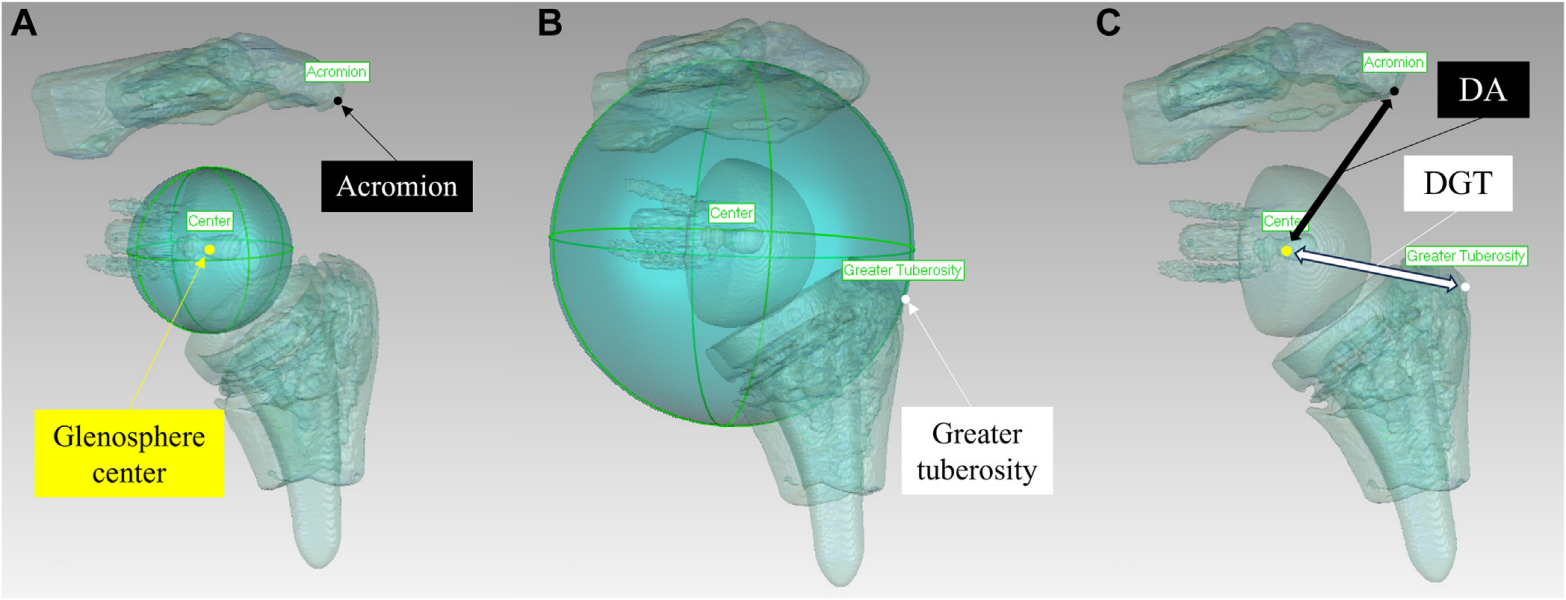
(**A**) The glenosphere center and the most lateral point on the undersurface of the acromion in the true anteroposterior view of the glenosphere was determined with three-dimensional surface models (Geomagic Wrap; 3D Systems, Rock Hill, SC, USA). (**B**) The lateral tip of the greater tuberosity was determined by reference to the outer circle with the glenosphere center as its center, passing through the most lateral edge of the greater tuberosity. (**C**) The distance between the center of the glenosphere and the acromion (DA) and the distance between the center of the glenosphere and the greater tuberosity (DGT) were measured.

DA and DGT were measured with 3D surface models derived from computed tomography scans taken with the shoulder at the side position (Geomagic Wrap; 3D Systems, Rock Hill, SC, USA). Measurements were defined by utilizing 3D measurements based on a previous report to define the most lateral edge of the greater tuberosity and the most lateral part of the acromion.^12^ DA was defined as the distance between the glenosphere center and the most lateral point on the undersurface of the acromion in the true anteroposterior view of the glenosphere (Fig. 3). DGT was defined as the distance between the center of the glenosphere and the lateral tip of the greater tuberosity. The lateral tip of the greater tuberosity was determined by reference to the outer sphere with the glenosphere center as its center, just passing through the most lateral edge of the greater tuberosity.

Two board certified orthopedic surgeons (I.K. and K.K.) independently measured DA and DGT. The intraclass correlation coefficient was assessed, and the mean values were taken for the analysis. Thus, patients were assigned to one of two groups according to the measurement of 3D surface models: shoulders with DA ≥ DGT i.e. (Δ) = (DA) – (DGT) ≥ 0 or shoulders with DA < DGT i.e. (Δ) = (DA) – (DGT) < 0.

### Statistical analysis

All statistical analyses were performed with EZR (Saitama Medical Center, Jichi Medical University, Saitama, Japan), which is a graphical user interface for R (The R Foundation for Statistical Computing, Vienna, Austria). More precisely, it is a modified version of R commander designed to add statistical functions frequently used in biostatistics.

All analyses were performed after confirming normal distribution with the Kolmogorov-Smirnov test. The repeated measures analysis of variance with post hoc Bonferroni correction was used to compare the preoperative and postoperative active range of motion and Constant scores in each group. Student’s *t* test and Fisher’s exact test were performed to compare other data between the groups. In order to assess intraclass correlation of measurements, the intraclass correlation coefficient agreement for measurements were used. *P* values < 0.05 were considered statistically significant. A post hoc power analysis using an α value of 0.05 was performed to compare the mean closest distance between the greater tuberosity and the acromion among the two groups.

## Results

**Figure 4:**
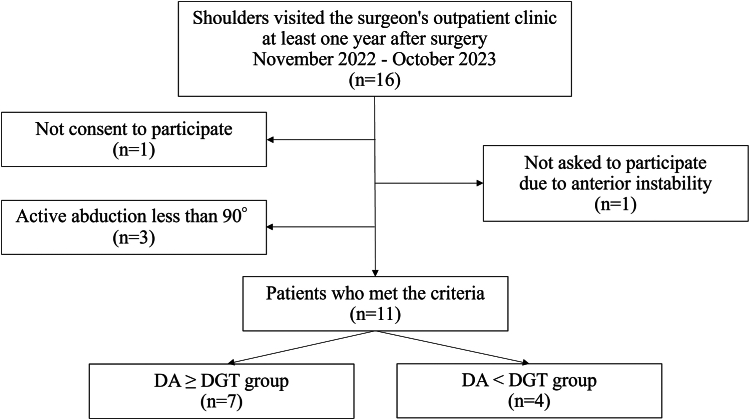
Flowchart of the patient selection *DGT*, distance between the glenosphere center and the greater tuberosity; *DA*, distance between the glenophere and the acromion.

16 shoulders in 15 patients visited the surgeon's outpatient clinic at least one year after surgery (Fig. 4). A total of 5 patients (5 shoulders) were excluded: 1 patient did not consent to participate in the study; 1 patient was not asked to participate by the surgeon due to a complication of anterior instability; and 3 patients had an active abduction range of motion of less than 90°. Finally, this study evaluated 11 shoulders of 10 patients. The patients consisted of 3 men and 7 women with a mean age of 76 years (range, 46-85). There were 7 shoulders with DA ≥ DGT, and 4 shoulders with DA < DGT. Sex, affected side, mean age at surgery, body weight, and body mass index were similar between the groups (Table I).

**Table I:** Demographic data of each group.

	DA ≥ DGT n = 7	DA < DGT n = 4	*P* value
Male/female	3/4	0/4	.2363
Right/left	3/4	4/0	.1939
Dominant/nondominant	3/4	4/0	.1939
Age, year	75 ± 14	79 ± 5	.6665
Body weight, kg	55 ± 15	57 ± 16	.8737
Height, cm	155 ± 13	153 ± 6	.6981
BMI, kg/m^2^	22.4 ± 3.0	24.3 ± 6.3	.5103

*BMI*, body mass index; *DGT*, distance between the glenosphere center and the greater tuberosity; *DA*, distance between the glenophere and the acromion.

The values are given as mean ± standard deviation.

The humeral prostheses used in all shoulders had a 145° neck-shaft angle. A 36-mm glenosphere was used in 9 shoulders and a 39-mm glenosphere was used in 2 shoulders (Table II). The thickness of polyethylene inserts was +0 mm in 10 shoulders, and +3 mm in 1 shoulder. The retroversion of the humeral implant was 20° in all shoulders. There was no significant difference in implant configuration between the groups.

**Table II:** Implant information of each shoulder.

	Shoulder number	Glenosphere, mm	Baseplate, mm	Polyethylene insert	Humeral stem size
DA ≥ DGT	No. 1	36	27	0	7
	No. 2	36	24.5	0	7
	No. 3	39	27	0	9
	No. 4	36	27	0	9
	No. 5	36	27	0	8
	No. 6	36	27	0	11
	No. 7	39	27	0	10
	Mean ± SD	37 ± 1	26.6 ± 0.9	2 ± 3	9 ± 2
DA < DGT	No. 8	36	24.5	0	8
	No. 9	36	27	3	9
	No. 10	36	27	0	9
	No. 11	36	27	0	8
	Mean ± SD	36 ± 0	26.4 ± 1.3	4 ± 3	9 ± 1
*P* value between the groups		.2821	.6953	.2932	.7932

*SD*, standard deviation; *DGT*, distance between the glenosphere center and the greater tuberosity; *DA*, distance between the glenophere and the acromion.

The diagnoses were as follows: irreparable massive rotator cuff tear/cuff tear arthropathy, 6 shoulders; primary osteoarthritis arthritis, 3 shoulders; revision after hemiarthroplasty, 2 shoulders (Table Ⅲ). There were no significant differences in active anterior elevation, external rotation, and internal rotation between the groups. Each postoperative active range of motion was significantly better than preoperative (*P* = .0014, *P* = .0063, *P* < .0001, respectively.) Although there were also no significant differences in ASES scores between the groups, postoperative ASES scores were significantly better than preoperative (*P* < .0001)

**Table III:** Clinical diagnosis, active range of motion, and American Shoulder and Elbow Surgeons Standardized Shoulder Assessment Form scores of each shoulder.

Group	Shoulder number	Diagnosis	Anterior elevation, degree		External rotation, degree		Internal rotation, degree		ASES score	
			Preoperative	Postoperative	Preoperative	Postoperative	Preoperative	Postoperative	Preoperative	Postoperative
DA ≥ DGT	No. 1	Primary OA	95	130	−10	15	Buttocks	L3	33	70
	No. 2	Irreparable RCT	80	140	10	20	L4	L3	47	85
	No. 3	Irreparable RCT	90	140	20	30	L5	L3	26	98
	No. 4	Revision after hemiarthroplasty	100	140	20	10	L5	L3	2	87
	No. 5	CTA	100	130	−10	15	L3	L3	43	95
	No. 6	Primary OA	90	120	0	10	Sacrum	L4	2	92
	No. 7	Massive RCT	10	130	0	15	L5	L4	13	93
	Mean ± SD		81 ± 32	133 ± 8	4 ± 13	16 ± 7	L5 ± 1	L3 ± 0	24 ± 19	89 ± 9
DA < DGT	No. 8	Irreparable RCT	30	120	20	40	Buttocks	L2	22	80
	No. 9	Primary OA	80	130	10	10	Buttocks	L5	39	87
	No. 10	Revision after hemiarthroplasty	40	120	−20	10	Buttocks	L3	2	87
	No. 11	Irreparable RCT	140	130	10	20	L3	L2	45	92
	Mean ± SD		73 ± 50	125 ± 6	5 ± 17	20 ± 14	Sacrum ± 2	L3 ± 1	27 ± 19	87 ± 5
*P* value for the groups (DA ≥ DGT or DA < DGT)			.5570		.7580		.7522		.9253	
*P* value for the time (preoperative or postoperative)			**.0014**		**.0063**		**>.001**		**>.001**	

*SD*, standard deviation; *OA*, osteoarthritis; *RCT*, rotator cuff tear; *CTA*, cuff tear arthropathy; *ASES score*, American Shoulder and Elbow Surgeons Standardized Shoulder Assessment Form score; *DGT*, distance between the glenosphere center and the greater tuberosity; *DA*, distance between the glenophere and the acromion.

Bold text indicates a significant *P* value.

Measurement of DA and DGT by two observers were similar (Table Ⅳ). The intraclass correlation coefficients for measuring DA and DGT were 0.989 and 0.978, respectively.

**Table IV:** Distance measurement data of each shoulder.

	Shoulder number	DA, mm		DGT, mm		Δ = DA – DGT, mm	
		Observer 1	Observer 2	Observer 1	Observer 2	Observer 1	Observer 2
DA ≥ DGT	No. 1	45.8	44.7	42.0	41.3	3.8	3.4
	No. 2	35.0	36.3	33.6	33.8	1.4	2.5
	No. 3	46.6	46.1	40.6	41.8	6.0	4.3
	No. 4	48.6	49.8	36.7	37.7	11.9	12.2
	No. 5	41.8	40.2	38.5	37.4	3.3	2.8
	No. 6	44.5	44.6	38.6	38.6	5.9	6.0
	No. 7	44.5	43.4	40.5	41.0	4.0	2.4
DA < DGT	No. 8	38.7	39.8	49.5	48.0	−10.8	−8.2
	No. 9	40.1	40.1	41.1	41.1	−1.0	−1.0
	No. 10	25.8	25.0	36.5	35.4	−10.7	−10.4
	No. 11	35.6	35.4	37.7	37.7	−2.1	−2.2
	Mean ± SD	40.6 ± 6.6	40.5 ± 6.7	39.6 ± 4.1	39.4 ± 3.8	1.1 ± 6.9	1.1 ± 6.4

*SD*, standard deviation; *DGT*, distance between the glenosphere center and the greater tuberosity; *DA*, distance between the glenophere and the acromion.

**Figure 5:**
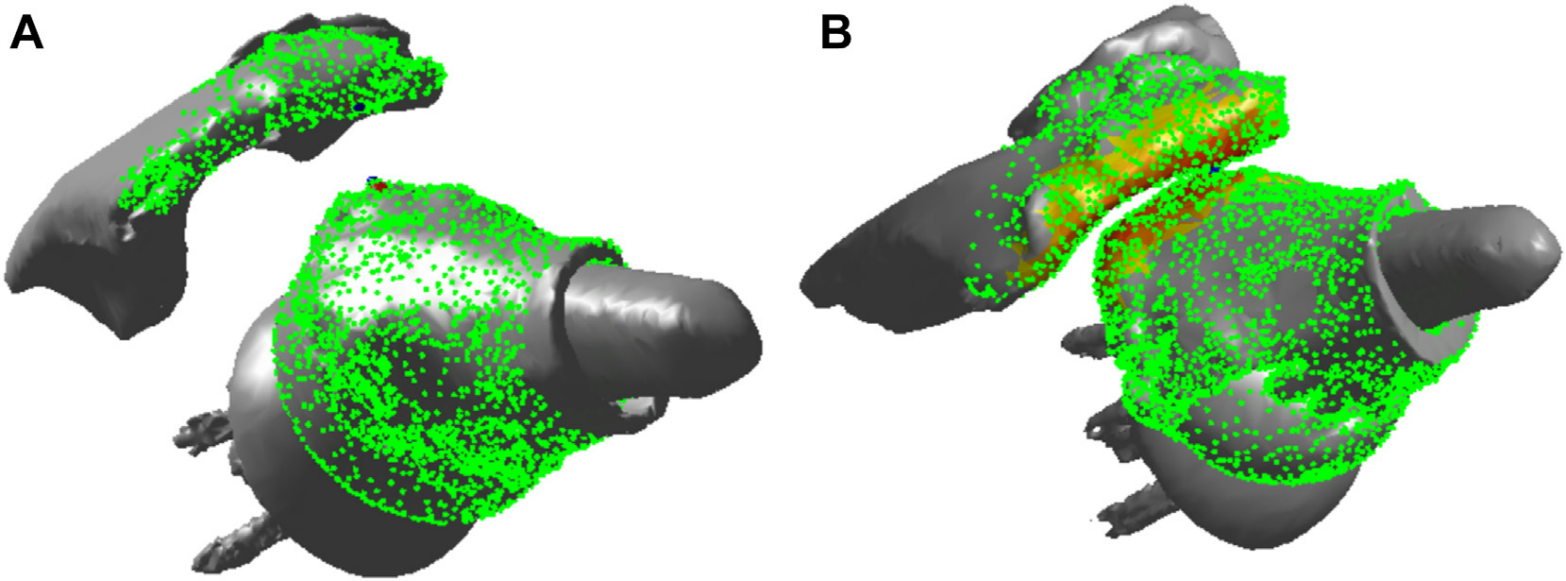
(**A**) Typical example of a shoulder with DA ≥ DGT. (**B**) Typical example of a shoulder with DA < DGT. *DGT*, distance between the glenosphere center and the greater tuberosity; *DA*, distance between the glenophere and the acromion.

**Figure 6:**
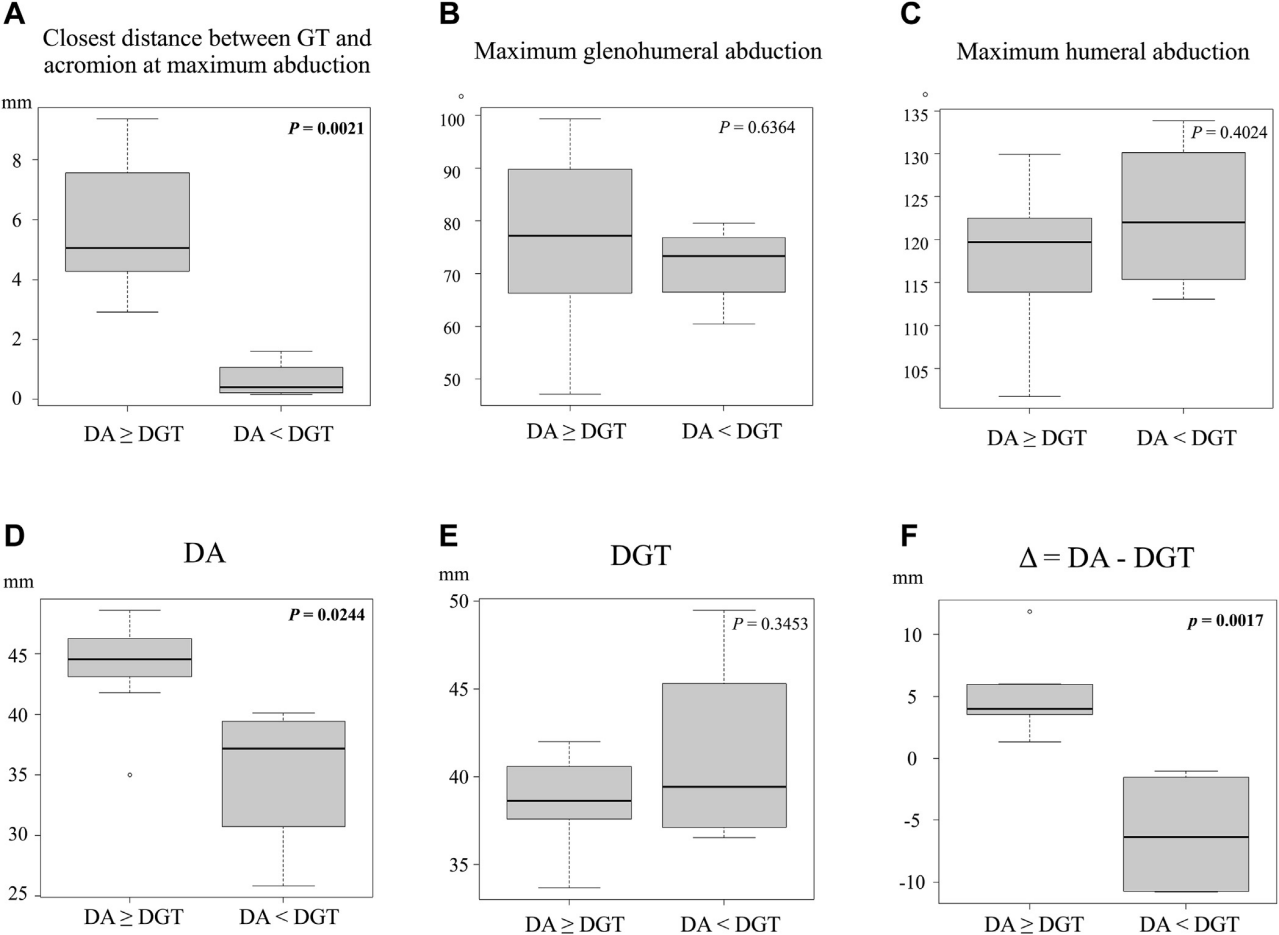
(**A**) The mean closest distance between the greater tuberosity (GT) and the acromion at maximum abduction in each group. (**B**) The mean maximum glenohumeral abduction angle in each group. (**C**) The mean maximum humeral abduction in each group. (**D**) The mean distance between the center of the glenosphere and the acromion (DA) in each group. (**E**) The mean distance between the center of the glenosphere and the greater tuberosity (DGT) in each group. (**F**) The mean (Δ) = (DA) – (DGT) in each group. Bars indicate the means and standard deviation (SD). Statistical significance was analyzed using student’s *t* test. Bold indicates statistically significant *P* values (*P* < .05).

Typical examples of the different patterns in both groups are shown in Figure 5. The closest distance between greater tuberosity and acromion at maximum abduction was significantly greater in shoulders with DA ≥ DGT than in those with DA < DGT (5.9 ± 2.4 mm vs. 0.6 ± 0.7 mm, respectively, *P* = .0021) (Fig. 6). The post hoc power analysis indicated that the power was 1.0. There were no significant differences in maximum glenohumeral abduction angle and humeral abduction angle between the two groups (76.5° ± 18.6° vs. 71.7° ± 8.0° respectively, *P* = .6364, and 117.7° ± 9.2° vs. 122.8° ± 9.2°, respectively, *P* = .4024). DA was significantly greater in shoulders with DA ≥ DGT than in those with DA < DGT (43.7 ± 4.4 mm vs. 35.1 ± 6.7 mm, respectively, *P* = .0275). However, there was no significant difference in DGT between the two groups. (Δ) = (DA) – (DGT) was significantly greater in shoulders with DA ≥ DGT than in those with DA < DGT (5.0 ± 3.4 mm vs. −5.8 ± 4.9 mm, respectively, *P* = .0019).

## Discussion

This study demonstrated that when DGT is less than DA in shoulders with RSA, the closest distance between the greater tuberosity and the acromion at maximum abduction is significantly wider compared to cases where DGT is greater than DA by three-dimensional measurement. This result would suggest acromial impingement or subacromial notching is less likely to occur in shoulders with RSA when DA is greater than DGT.

Maintaining a gap between the proximal humerus and acromion appears advantageous in preventing subacromial notching or impingement, thereby preserving postoperative clinical outcomes.^3^ We hypothesized that if DA is greater than DGT, the gap between the greater tuberosity and the acromion is wide enough to avoid impingement on the acromion. This hypothesis was validated in our study. To avoid subacromial impingement or notching, it might be important to keep DGT smaller than DA or to make DA larger than DGT.

Adequate humeral lateralization by humeral side appears beneficial for enhancing RSA outcome to reduce scapular notching and to improve external rotation,^8^^,^^18^ but it may result in a larger DGT. The humerus is usually externally rotated during abduction,^24^ and there are possibilities that the distance between the acromion and humerus at abduction is influenced by the degree of this rotation, and that glenohumeral external rotation could compensate to some extent for a large DGT to maintain the separation distance. In fact, there was no significant difference in DGT between the groups, despite a significant difference in DA between them.

In order to keep DA larger than DGT, maintaining sufficient gap between the greater tuberosity and the acromion, a larger DA may be more critical than a smaller DGT. One method to achieve a larger DA is through inferior glenosphere placement. Inferior glenosphere placement has been reported to reduce scapular notching,^9^ potentially decreasing the occurrence of both subacromial and scapular neck impingement. However, caution is warranted with inferior glenosphere placement that leads to humeral distalization, as scapular spine fractures after RSA have been reported to be associated with humeral distalization.^2^ Another way to achieve a larger DA is to medially position the glenosphere center, ensuring a distance from the lateral edge of the acromion. Glenoid lateralization has been associated with higher strains on the scapular spine and an increased rate of acromial fractures after RSA.^5^^,^^7^ Therefore, inferior and medial glenosphere placement might be considered to increase DA while avoiding other complications. However, it's also essential to exercise caution with medialized glenosphere placement due to its possible association with scapular notching.^4^

Shoulders lacking abduction of at least 90 degrees were excluded from this study. We considered the distance between the greater tuberosity and the acromion decreased as the abduction angle increased, so having shoulders with similar abduction angles is important. We observed no significant difference in both glenohumeral and humeral abduction angle between the two groups, and conclude our findings were not influenced by variations in arm elevation.

This study had several limitations. First, DA and DGT were measured with 3D surface models and complex software. Practical extrapolation of our findings will require evidence that 2D measures from X-ray images substantially reproduce our 3D measures of DA and DGT. Furthermore, since X-ray evaluation was not conducted in this study, it remains unclear which X-ray parameters may influence DA and DGT in detail, necessitating further research in this regard. Second, the study involved a limited sample size, but had sufficient power, potentially limiting the generalizability of the findings. Larger and more diverse cohorts may be needed to enhance the external validity of the results. Third, the present study investigated the likelihood of acromial impingement occurring as basic research. In the future we plan on a clinical study with and increased number of patients. Fourth, the study utilized a specific implant system, potentially limiting the generalizability of the findings to other implant brands or designs. Variability in implant characteristics may influence the observed outcomes. Fifth, although the modeling accuracy has been confirmed with a root mean square error of 0.4 mm^15^, it's possible that the accuracy has affected findings ranging from 0 to 10 mm in this study. Similarly, while the matching method's accuracy was 0.5mm for in-plane translation and 0.8° for in-plane rotation in a previous study,^14^ the accuracy might have influenced the result. Finally, variation in subjects, such as sex, age, height, or operative indications, could affect shoulder kinematics and the results in this study. The average height of the subjects was 154 cm, and this very short stature might result in particularly short distances.

## Conclusion

When DGT is less than DA in shoulders with RSA, the closest distance between the greater tuberosity and the acromion at maximum abduction is significantly wider compared to cases where DGT is greater than DA by three-dimensional measurement. Therefore, acromial impingement or subacromial notching is less likely to occur in shoulders with RSA when DA is greater than DGT. To avoid acromial impingement, it might be important to make DA larger than DGT.

## Disclaimers:

Funding: Research grant support from the Medacta International was received in partial support of this work. The funding agency played no role in the study design, recruitment of subjects, interpretation of data, or preparation of this manuscript.

Conflicts of interest: Itaru Kawashima is supported by The Uehara Memorial Foundation Overseas Postdoctoral Fellowships and by The Nakatomi Foundation. Norimasa Takahashi is a paid consultant of Medacta International. Kenji Kitamura is supported by The Uehara Memorial Foundation Overseas Postdoctoral Fellowships. Thomas W. Wright is a consultant and receives royalties from Exactech, Inc. Scott A. Banks is a consultant and receives royalties from Enovis and Stryker. The other authors, their immediate families, and any research foundation with which they are affiliated have not received any financial payments or other benefits from any commercial entity related to the subject of this article.
